# Supplementary material for: Substrate binding plasticity revealed by Cryo-EM structures of SLC26A2
Source: Nat Commun. 2024 Apr 29;15:3616. doi: 10.1038/s41467-024-48028-3 (PMC11059360; doi:10.1038/s41467-024-48028-3)
Supplement: Supplementary file 1 — Supplementary Information [file 41467_2024_48028_MOESM1_ESM.pdf]

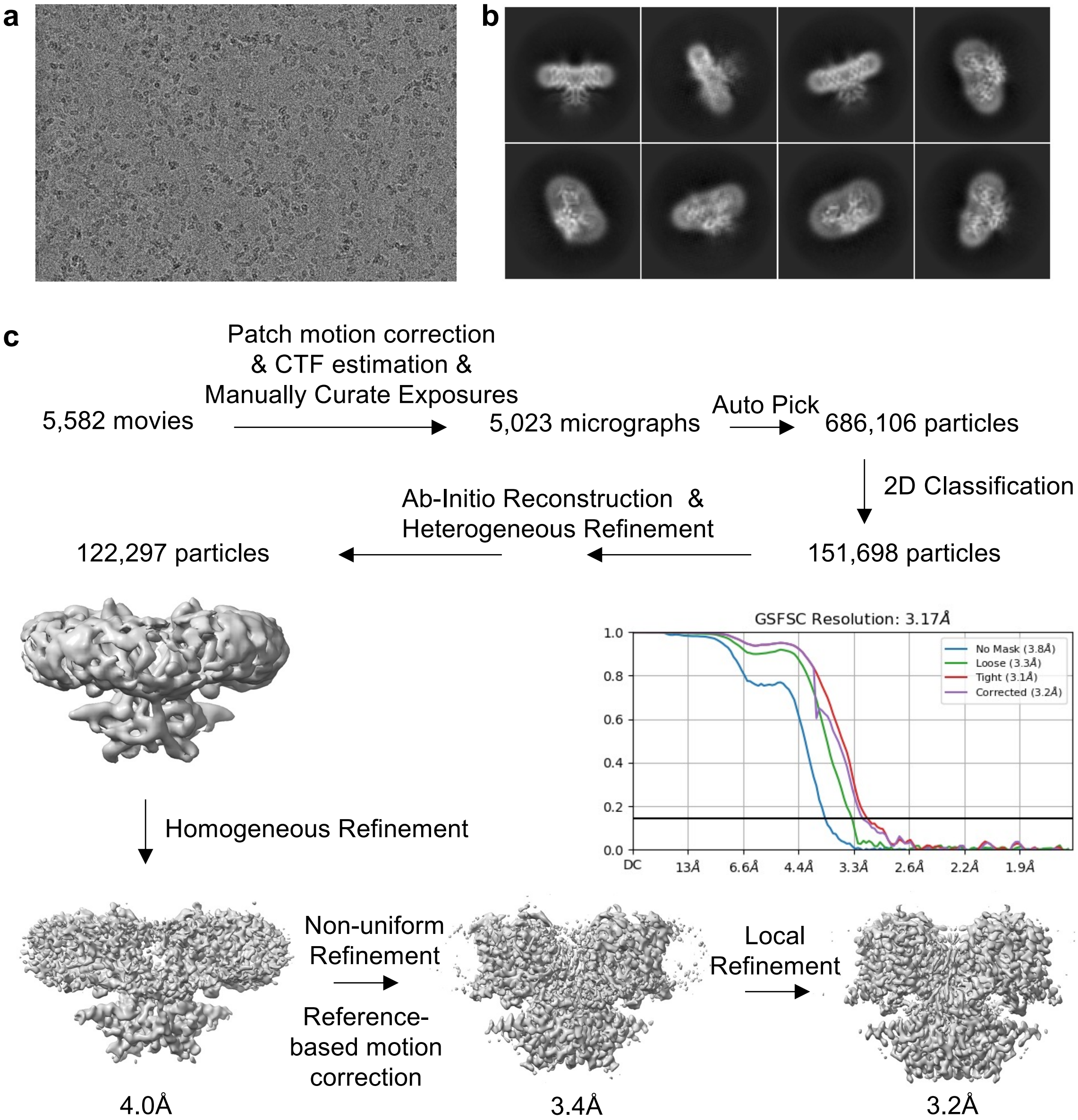

Supplementary Fig. 1: Cryo-EM data processing workflow for SLC26A2-Cl$^-$. a. A representative cryo-EM micrograph. **b.** Selected 2D averages. **c.** The data processing flowchart in cryoSPARC leads to a final map at ~3.2Å resolution demonstrated by the gold-standard FSC.

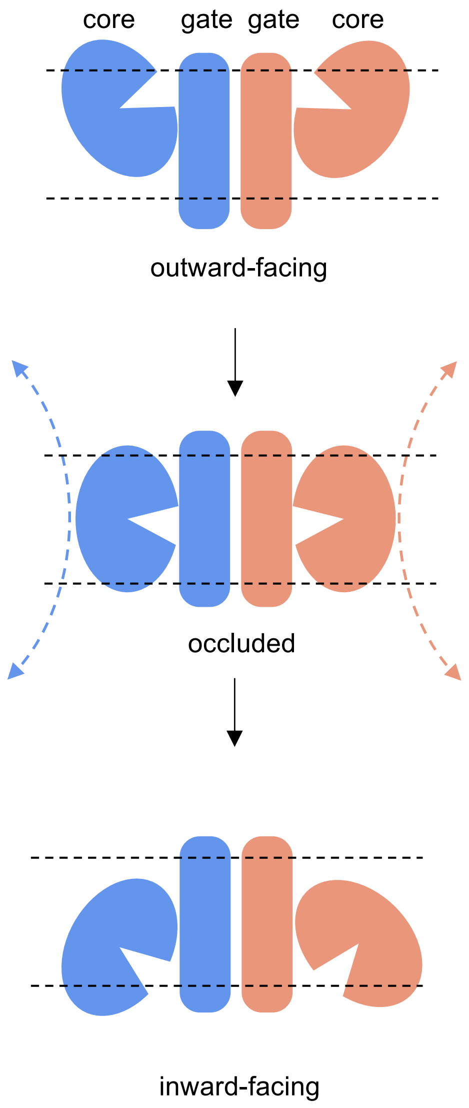

Supplementary Fig. 2: Hypothetical elevator-like movement within SLC26A2. The cartoon representation of the gate and core domains is viewed from the side of the membrane bilayer (dashed lines)**.** The core domain moves up and down in the bilayer to change the transporter’s conformation among outward-facing, occluded, and inward-facing.

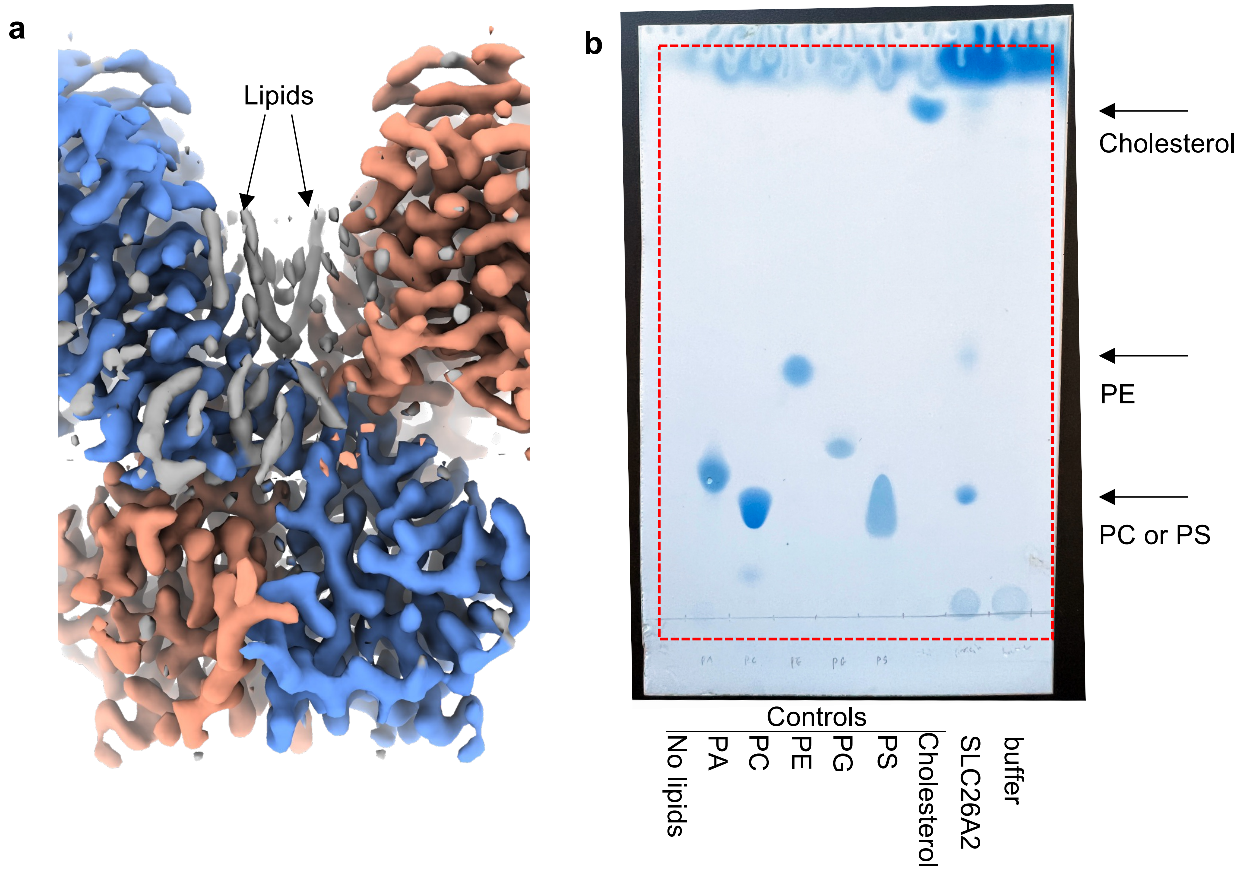

Supplementary Fig. 3: Lipid analysis of purified SLC26A2. a. Sausage-like lipid densities in the cryo-EM map are colored in gray. The view is the same as in Figure 1A. **b.** uncropped thin-layer chromatography to identify the composition of lipids. From left to right, lanes 1~7 are various controls; lane 8 is the purified SLC26A2, and lane 9 is the buffer used for protein purification.

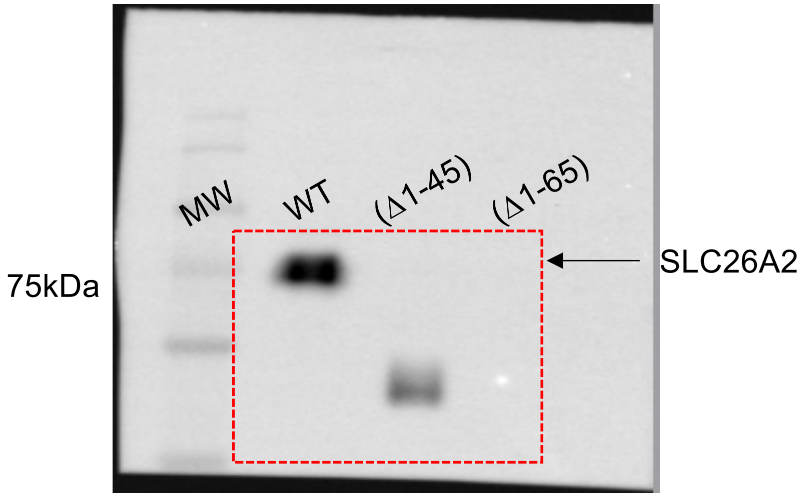

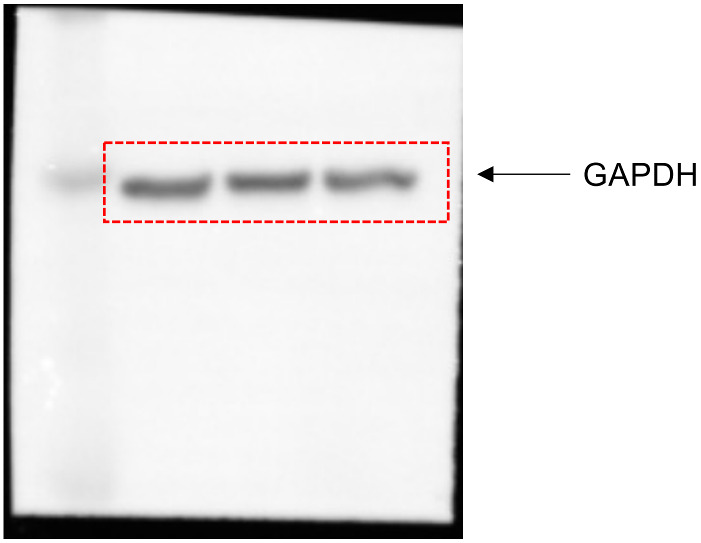

Supplementary Fig. 4: Expression level of SLC26A2 N-terminal truncations. From left to right, the lanes are molecular weight, wild-type, Δ1-45, and Δ1-65.

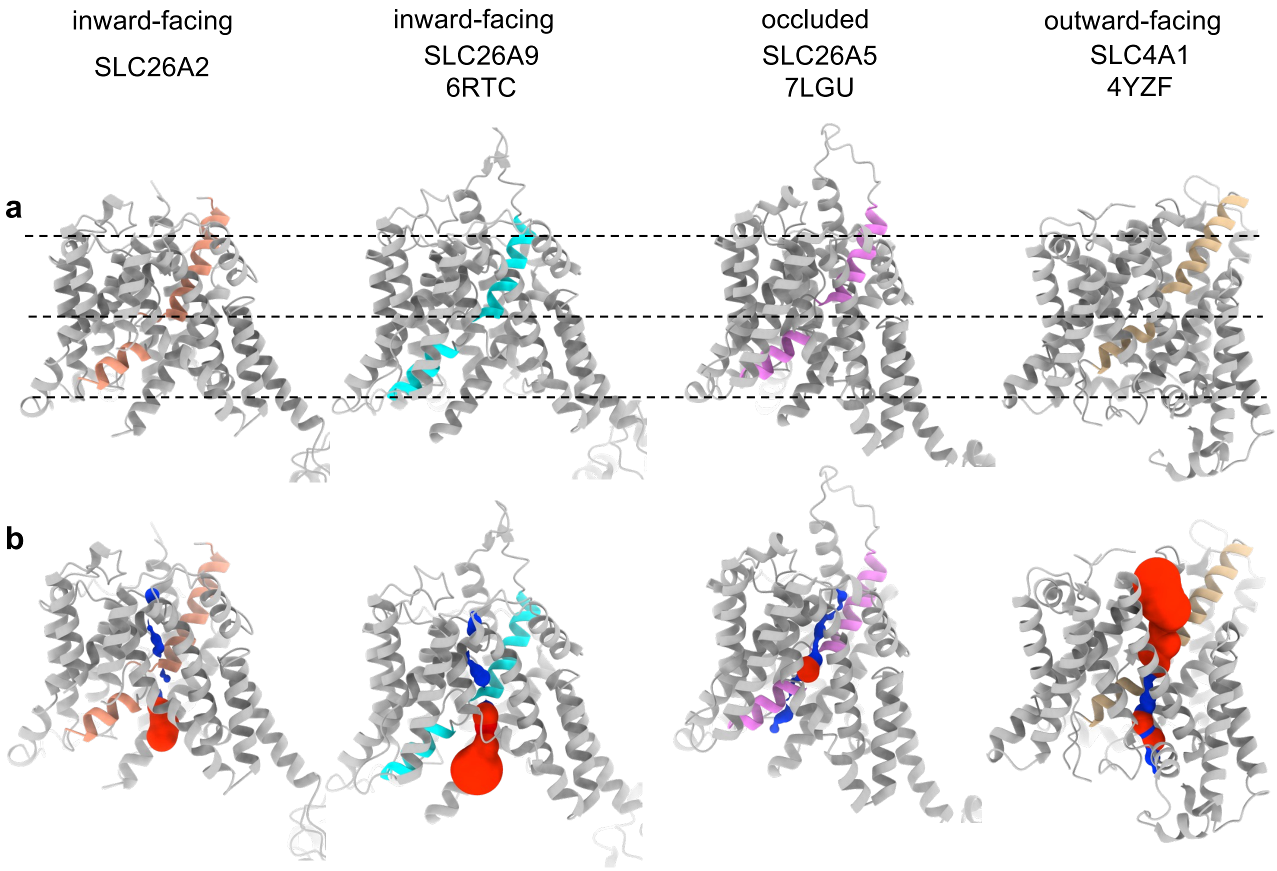

Supplementary Fig. 5: Comparison of SLC homologs. a. The vertical position of TM3/TM10 is shown in every protomer. **b.** The substrate-translocation pathway i s shown in every homolog. It is colored red when the radius is larger than 1.8Å and blue when it is smaller than 1.8Å.

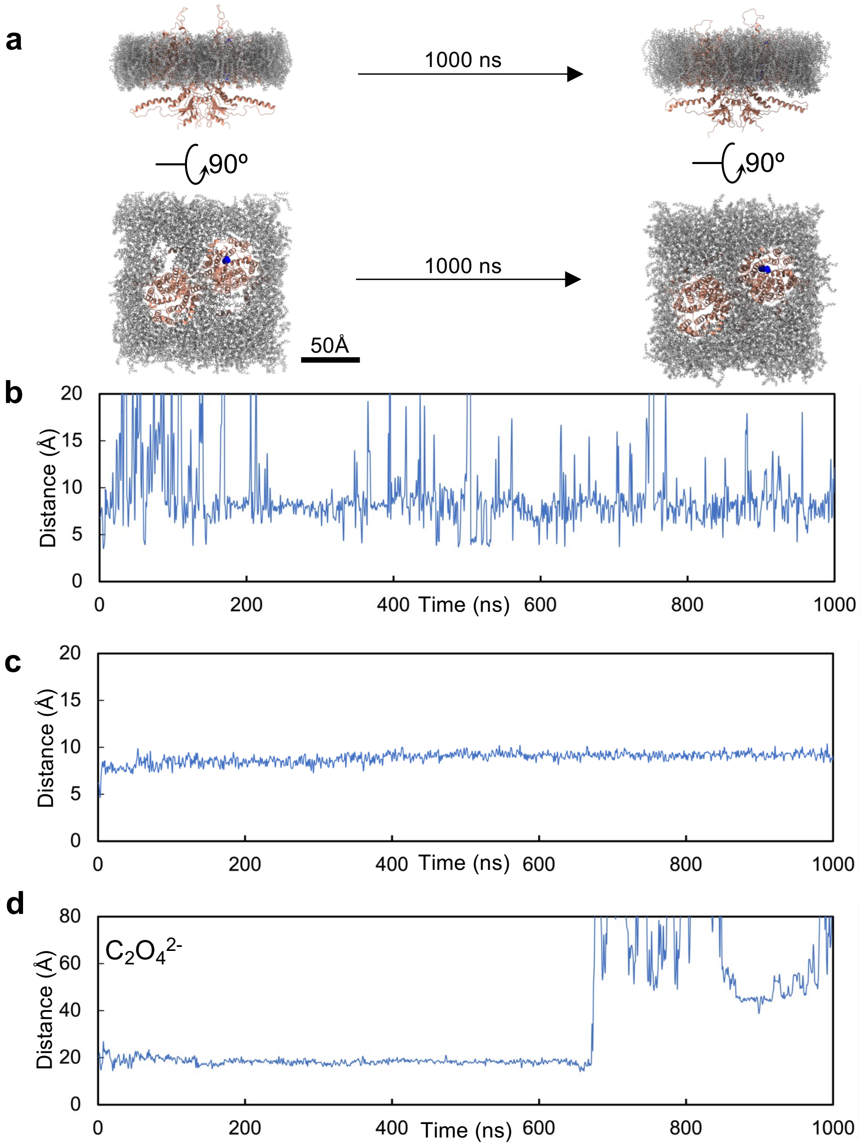

Supplementary Fig. 6: Substrate stability analyzed by molecular dynamics simulations. a. Representative snapshots of the system from the beginning and end of MD simulation. Lipids are in grey, protein is in dark salmon, and the calculated substrate-translocation pore is in blue. The temporal changes in the distance between the Cα of G166 and corresponding substrates are plotted during the 1 μs representative simulations of SLC26A2-Cl^- (**b**), SLC26A2-SO_4^{2-} (**c**), and SLC26A2-$C_2O_4^{2-}$ (**d**).

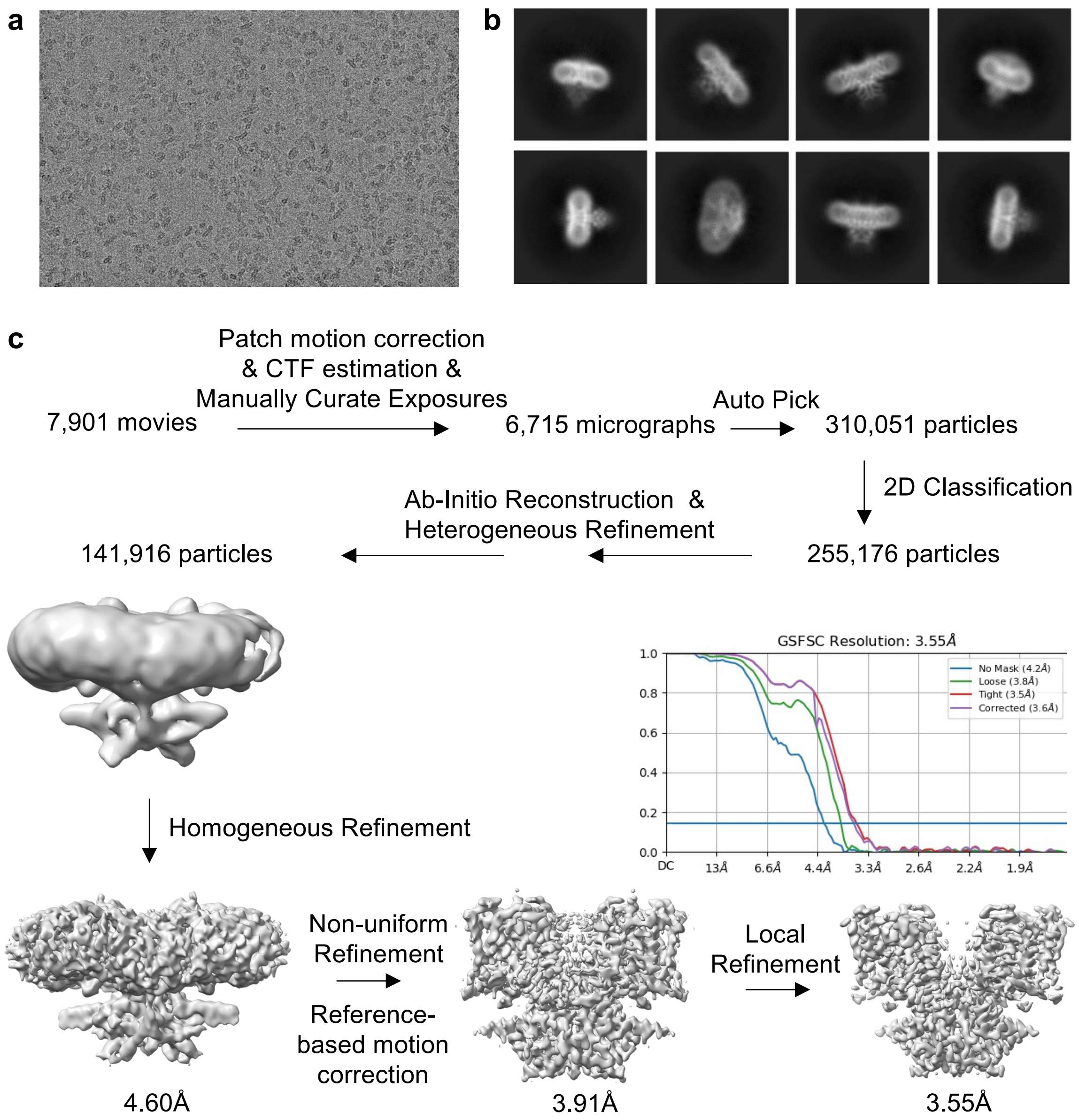

Supplementary Fig. 7: Cryo-EM data processing workflow for SLC26A2-SO_4^{2-}. a. A representative cryo-EM micrograph. **b.** Selected 2D averages. **c.** The data processing flowchart in cryoSPARC leads to a final map at ~3.6Å resolution demonstrated by the gold-standard FSC.

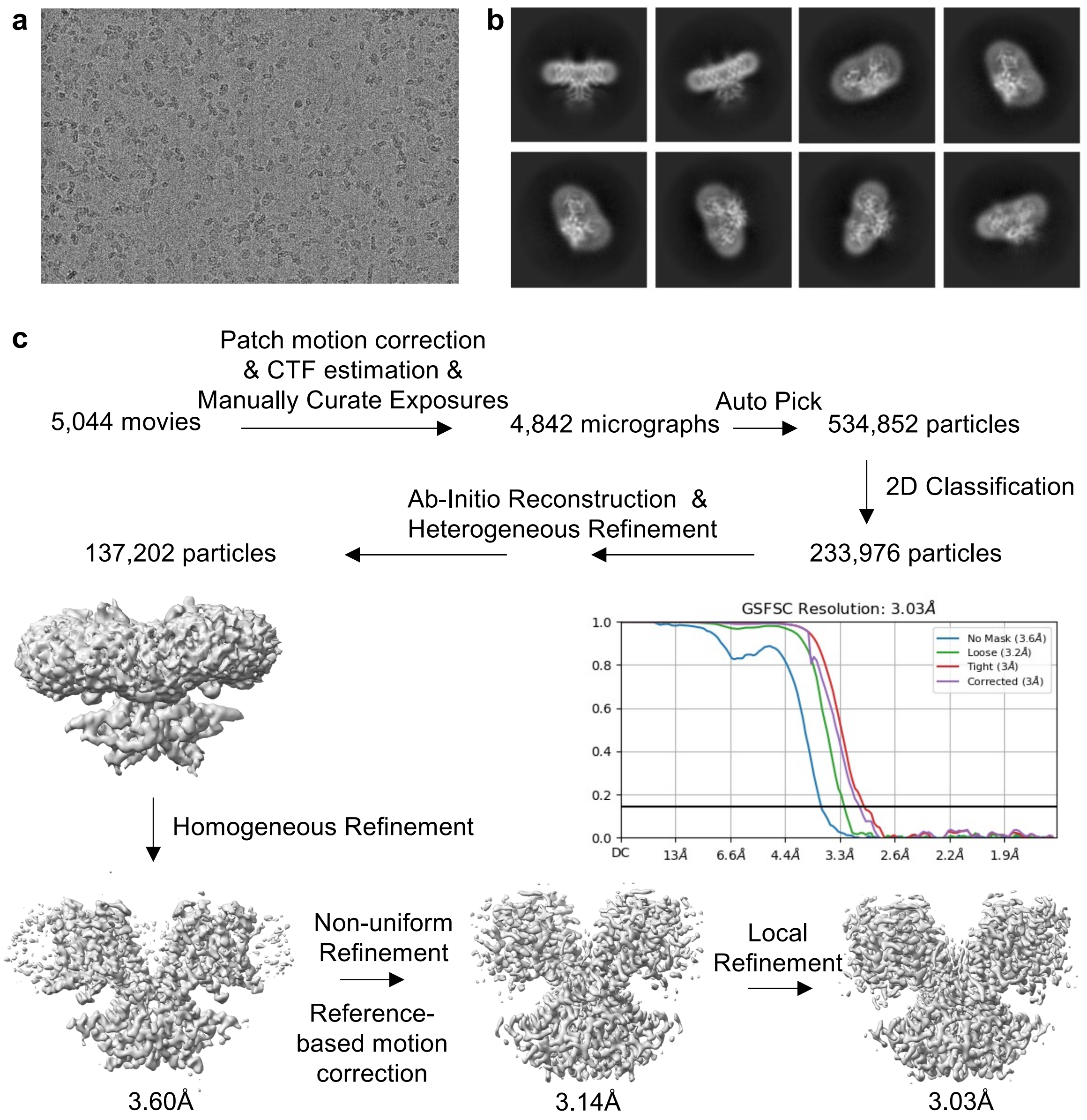

Supplementary Fig. 8: Cryo-EM data processing workflow for SLC26A2-$C_2O_4^{2-}$. a. A representative cryo-EM micrograph. **b.** Selected 2D averages. **c.** The data processing flowchart in cryoSPARC leads to a final map at ~3Å resolution, demonstrated by the gold-standard FSC.

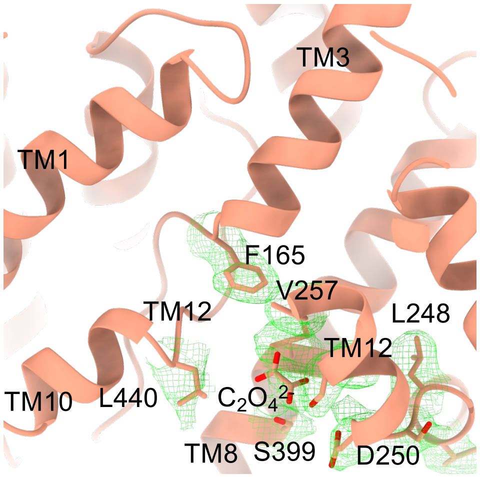

Supplementary Fig. 9: Substrates binding. The distinct binding site for $C_2O_4^{2-}$ molecule.

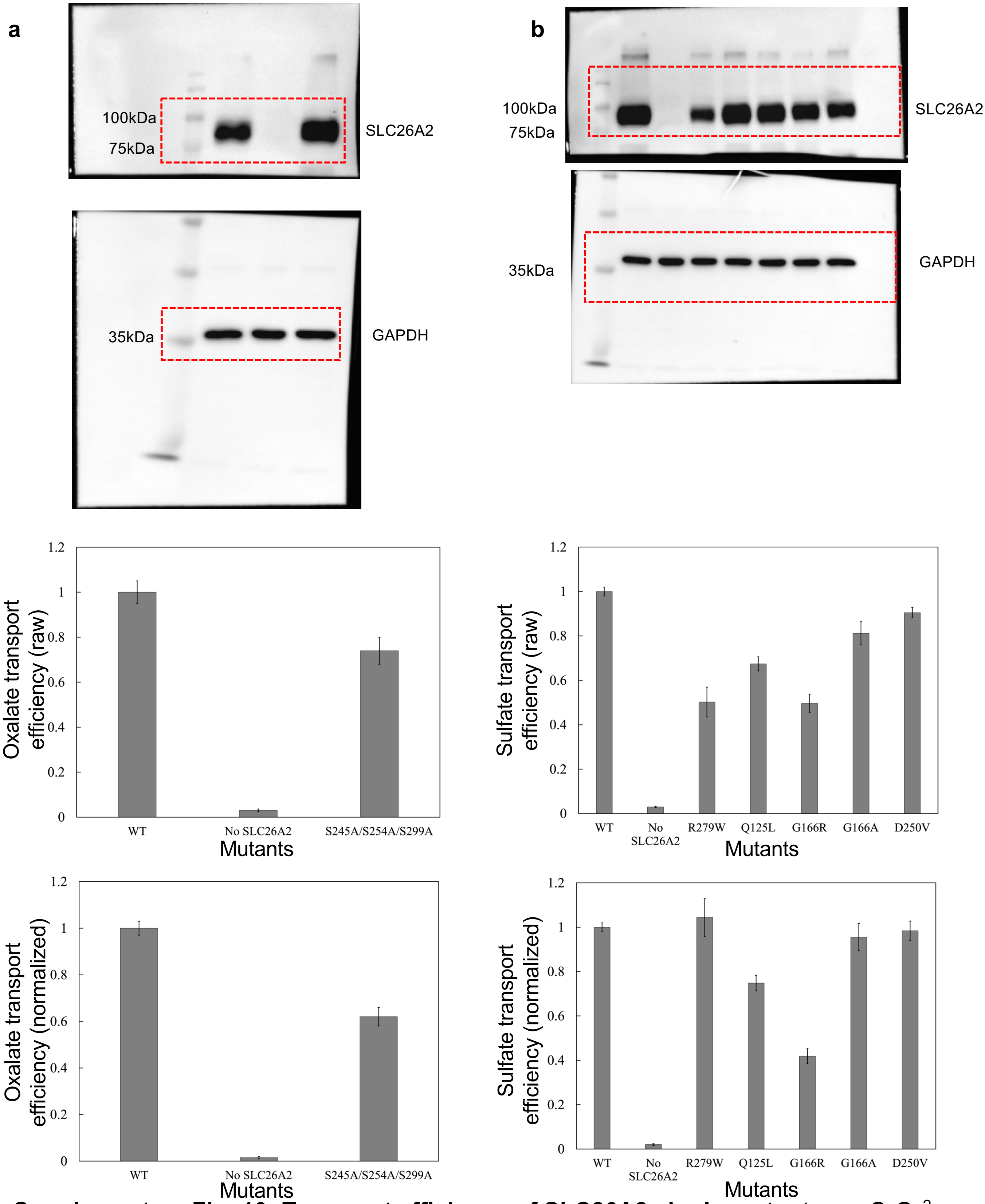

Supplementary Fig. 10: Transport efficiency of SLC26A2 single mutants. a. $C_2O_4^{2-}$. **b.** SO_4^{2-}. From top to bottom, the panels represent uncropped western blots showing mutant protein expression, raw transport efficiency, and transport efficiency normalized to the expression level. GAPDH is the control protein for the blots. The lane order corresponds to the sequence presented in the graphs. The efficiency and error bars were calculated from independent cellular transport experiments provided in the source data.

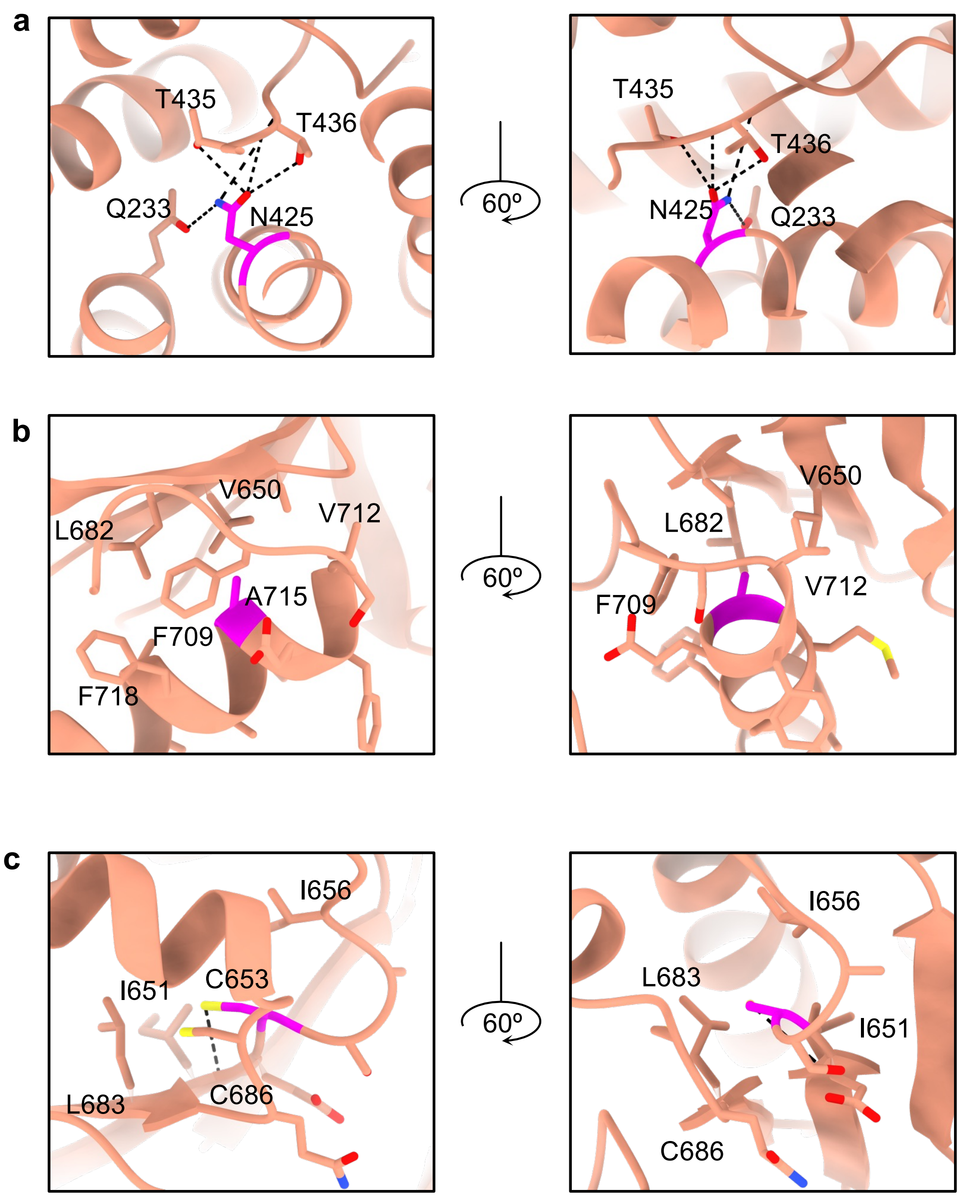

Supplementary Fig. 11: Pathologically relevant residues. a. The H-bond network around N425. **b**. The hydrophobic local environment around A715. **c**. Residues surrounding C653.

a

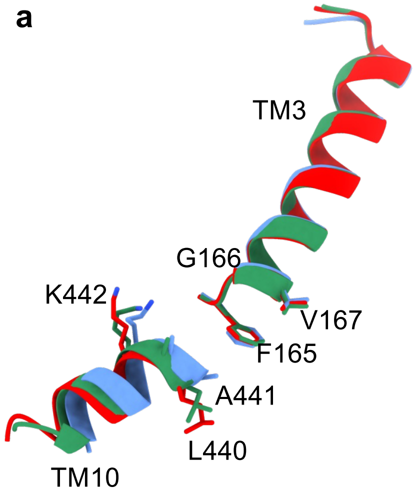

	F165/K442	G166/A441	V167/L440
Cl^-	9.2 ± 0.4	9.8 ± 0.7	11.3 ± 1.0
SO_4^{2-}	8.8 ± 0.4	9.4 ± 0.5	10.3 ± 1.0
$C_2O_4^{2-}$	9.8 ± 0.6	9.8 ± 0.6	12.2 ± 1.2

b

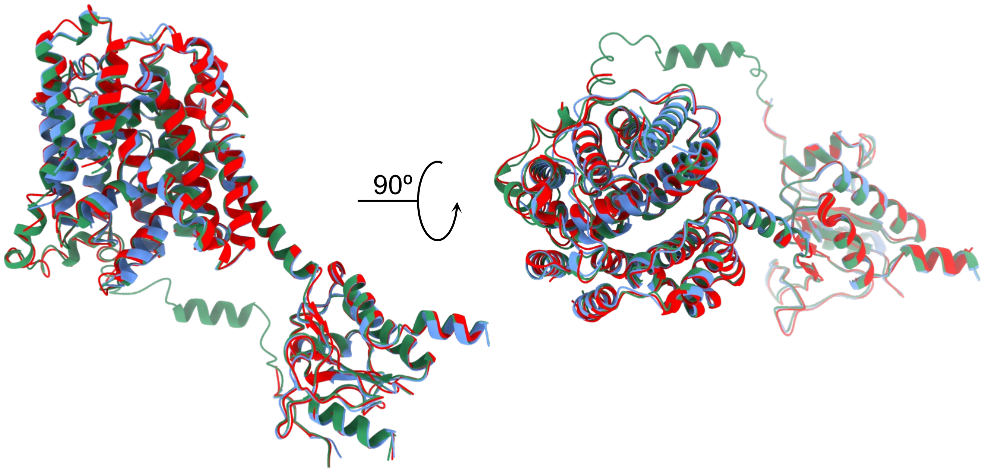

Supplementary Fig. 12: Comparison of SLC26A2 structures. a. Three pairs of residues are used to gauge the substrate-binding pocket size with their distances (Å) shown in the table. **b**. The superposition of all three SLC26A2 structures shows no significant global change. SLC26A2-Cl^-: green; SLC26A2-SO_4^{2-}: blue; SLC26A2-$C_2O_4^{2-}$: red.

Supplementary Table 1: Cryo-EM data collection, model refinement, and validation statistics

	SLC26A2, Cl^-	SLC26A2, $C_2O_4^{2-}$	SLC26A2, SO_4^{2-}
**Data Collection and Processing**			
Microscope	Titan Krios	Titan Krios	Titan Krios
Voltage (kV)	300	300	300
Magnification (nominal)	92,000	92,000	92,000
Electron Dose ($e^-/Å^2$)	60	60	60
Camera	Gatan K3	Gatan K3	Gatan K3
Defocus range (um)	-1 ~ -2.5	-1 ~ -2.5	-1 ~ -2.5
Pixel size (Å)	0.826	0.826	0.826
Movies collected	5582	5044	7901
Symmetry imposed	C2	C2	C2
Initial particle images (no.)	686,106	534,852	310,051
Final particle images (no.)	122,297	137,202	141,916
Map resolution (Å)	3.17	3.03	3.55
FSC cutoff	0.143	0.143	0.143
Map resolution range	2.8 ~ 9.6	2.7 ~ 9.3	2.9 ~ 9.8
Sharpening B-factor ($Å^2$)	-133.7	-147.2	-142.1
**Refinement statistics**			
Initial model used	*de novo*	*de novo*	*de novo*
Model resolution (FSC = 0.5)	3.10	3.16	3.61
Correlation Coefficient (Mask)	0.85	0.85	0.79
Model composition			
Non-hydrogen atoms	8872	8232	8022
Protein residues	1210	1152	1090
Ligands	2	4	2
R.m.s. deviations			
Bonds (Å)	0.003	0.003	0.004
Bond angles (º)	0.541	0.510	0.666
Validation			
MolProbity score	1.71	1.35	1.86
Clash score	7.37	1.35	10.44
Poor rotamers (%)	0.22	0.00	0.00
Ramachandran plot			
Favored (%)	95.64	98.76	95.31
Allowed (%)	4.36	1.24	4.69
Disallowed (%)	0	0	0
EMDB access code	41427	41428	41429
PDB access code	8TNW	8TNX	8TNY

Supplementary Table 2: Missense mutations in *SLC26A2*

Protein change	Conditions	Clinical significance
D111Y	Diastrophic dysplasia	Likely pathogenic
Q125L	Atelosteogenesis type II	Pathogenic
A133V	Diastrophic dysplasia	Pathogenic
S157P	Multiple epiphyseal dysplasia type 4	Likely pathogenic
G166R	Diastrophic dysplasia	Likely pathogenic
D250V	Diastrophic dysplasia	Likely pathogenic
R279W	Diastrophic dysplasia	Pathogenic/Likely pathogenic
C311R	Achondrogenesis, type IB, Diastrophic dysplasia, Atelosteogenesis type II, Multiple epiphyseal dysplasia type 4	Likely pathogenic
A386G	Atelosteogenesis type II, Achondrogenesis, type IB, Diastrophic dysplasia, Multiple epiphyseal dysplasia type 4	Likely pathogenic
A386V	Achondrogenesis, type IB, Atelosteogenesis type II, Diastrophic dysplasia, Multiple epiphyseal dysplasia type 4	Pathogenic
N425D	Diastrophic dysplasia, Achondrogenesis, type IB, Multiple epiphyseal dysplasia type 4, Atelosteogenesis type II	Pathogenic/Likely pathogenic
I426T	Atelosteogenesis type II, Achondrogenesis, type IB, Diastrophic dysplasia, Multiple epiphyseal dysplasia type 4	Likely pathogenic
I426N	Atelosteogenesis type II, Achondrogenesis, type IB, Diastrophic dysplasia, Multiple epiphyseal dysplasia type 4	Likely pathogenic
Q454P	Diastrophic dysplasia, broad bone platyspondylic variant	Pathogenic
A461V	Atelosteogenesis type II	Likely pathogenic
L483P	Sulfate transporter-related osteochondrodysplasia	Pathogenic
G484D	Diastrophic dysplasia	Likely pathogenic

W505R	Atelosteogenesis type II, Achondrogenesis, type IB, Diastrophic dysplasia, Multiple epiphyseal dysplasia type 4	Likely pathogenic
T512K	Diastrophic dysplasia, Achondrogenesis, type IB, Multiple epiphyseal dysplasia type 4, Atelosteogenesis type II	Pathogenic
S522F	Multiple epiphyseal dysplasia type 4	Pathogenic
C653G	Atelosteogenesis type II, Achondrogenesis, type IB, Diastrophic dysplasia, Multiple epiphyseal dysplasia type 4	Likely pathogenic
C653S	Connective tissue disorder, SLC26A2-Related Disorders, Diastrophic dysplasia, Achondrogenesis, type IB, Multiple epiphyseal dysplasia type 4, Atelosteogenesis type II, not provided, 3MC syndrome 2, Sulfate transporter-related osteochondrodysplasia, Achondrogenesis, type IB, Diastrophic dysplasia, Atelosteogenesis type II, Multiple epiphyseal dysplasia type 4	Pathogenic/Likely pathogenic
C653Y	Achondrogenesis, type IB, Atelosteogenesis type II, Diastrophic dysplasia, Multiple epiphyseal dysplasia type 4	Likely pathogenic
G678V	Osteochondrodysplasia, Multiple epiphyseal dysplasia type 4	Likely pathogenic
A715T	Atelosteogenesis type II, Achondrogenesis, type IB, Diastrophic dysplasia, Multiple epiphyseal dysplasia type 4	Likely pathogenic
