# Supplementary material for: Substrate binding plasticity revealed by Cryo-EM structures of SLC26A2
Source: Nat Commun. 2024 Apr 29;15:3616. doi: 10.1038/s41467-024-48028-3 (PMC11059360; doi:10.1038/s41467-024-48028-3)
Supplement: Supplementary file 3 — Description of Additional Supplementary Files [file 41467_2024_48028_MOESM3_ESM.pdf]

File name: Supplementary Movie 1
Description: The model of SLC26A2 fits into the 3.2Å resolution cryo-EM map.

File name: Supplementary Movie 2
Description: The N-terminal region of SLC26A2.

File name: Supplementary Movie 3
Description: The transmembrane region of SLC26A2.

File name: Supplementary Movie 4
Description: The STAS domain of SLC26A2.
